# Outcome of Acute Deep Venous Thrombosis Using Standard Treatment versus Thrombolytics: A Literature Review 

**Published:** 2019-10-01

**Authors:** Abdella Birhan, Tamrat Assefa, Alemseged Beyene, Pacifique Ndayishimiye, Minyahil Alebachew Woldu

**Affiliations:** 1 Department of Pharmacology and Clinical Pharmacy, School of Pharmacy, Addis Ababa University (AAU), Addis Ababa, Ethiopia; 2 Department of Clinical Pharmacy and Pharmacology, School of Pharmacy, Muhimbili Health and Allied Sciences (MUHAS), Dar Es Salaam, Tanzania

**Keywords:** Thrombolytic, Therapy, Deep venous thrombosis

## Abstract

Deep vein thrombosis (DVT) is a major health problem affectinga significant portion of population. Primary complications are Pulmonary Embolism (PE) in the short term and Post-Thrombotic Syndrome (PTS) in the long term. Thrombolytic drugs act by activating plasminogen which in turn forms the enzyme plasmin. Plasmin consequently degrades blood clots by breaking down the fibrin molecules which make up the clots help to degrade the already formed clot. They can be used using different route of administration, doses and durations. The purpose of this systematic review was to assess the outcome of thrombolytic therapy in terms of the efficacy, safety and effectiveness of the medicines.

Electronic searches of databases (MEDLINE and Google Scholar) were queried for articles written in English since 2000 GC. A total of 760 results were obtained using the search keys, and after excluding duplicates, 275 articles were selected. Finally, 9 randomized controlled trials (RCTs) which met the language of publication, study design and exclusion criteria were included in this systematic review.

The data were obtained from nine trials (6 countries), providing a study-level data of 1309 participants. Almost all studies revealed that thrombolytic treatment was effective in the management of acute DVT. In most of the studies, the rate of rethrombosis was lower in case of thrombolytic than standard management. Hence, addition of thrombolytic results in persistence and increases the clinical benefits. Thrombolytic therapy was very effective in reversing closed veins, in boosting the patency rate,whilereflux was higher in patients treated with anticoagulants.

Thrombolytic offers potential advantages over the standard treatment of DVT by reducing the proportion of patients with chronic disabling leg symptoms (such as PTS) by triple in the longer term. However, the incident of major bleeding was higher in patients receiving thrombolytics than anticoagulants.

## Introduction

 Deep Vein Thrombosis (DVT) is a major health problem affectinga significant portion of population. Primary complications are Pulmonary Embolism (PE) in the short term and Post Thrombotic Syndrome (PTS) in the long term^   1 ^. Standard treatment using propagation, but does not treat the occlusion itself ^   2 ^. However, over half of patients may suffer PTS in the long term, manifested by some degree of pain, swelling, skin pigmentation or venous ulceration of the affected leg in the follow up period of therapy despite of taking anticoagulants^   3 ^.

Elastic compression stockings had also been recommended by the American College of Chest Physicians Evidence Based Clinical Practice Guidelines as non-pharmacologic alternative for DVT patients to prevent PTS ^   4 ^. However, a meta-analysis (six random controlled trails including 1462 patients) recently indicates that elastic compression stockings are not sufficient to prevent PTS^   2 ^.

Thrombolytic drugs act by activating plasminogen which in turn forms the enzyme plasmin^   5 ^. Plasmin consequently degrades blood clots by breaking down the fibrin molecules which make up the clots to degrade clots already formed. They may be administered using different doses and durations as well as different route of administration. The theoretical advantage behind the loco/regional and catheter-directed methods is that they may reduce the necessary amount of thrombolytic (uses lower doses) and may reduce the risk of bleeding compared to systemic route^   6 ^.

A randomized trial comparing recombinant tissue plasminogen activator (rt-PA) versus anticoagulation alone demonstrated that 58%of the patients receiving rt-PA achieved greater than 50% clotlysis compared to 0% in those receiving anticoagulation alone and that rt-PA-treated patients had a trend toward reduced PTS if lysis was successful (56%vs 25%)^   7 ^. However, the incident of major bleeding was higher in patients receiving thrombolytic than anticoagulants^   8 ^.

The goals of therapy for acute DVT are minimizing the incidence of recurrent thrombosis, PE, decreasing the risk of chronic venous insufficiency and PTS in order to achieve the goals in which thrombolytic therapy plays a major role^   9 ^. Conventional anticoagulant therapy which aimed at the prevention of PE and recurrent venous thromboembolism (VTE) has been largely ineffective at treating PTS^   10 ^.

Current recommendation on treatment of iliofemoral venous thrombosis is percutaneous catheter-directed thrombolysis (CDT), either pharmacologic or pharmacomechanical as first-line therapy^   11 ^. Current reviews indicate that thrombolytic use increases the proportion of participants with any improvement in venous patency and complete clotlysis, and reduces the risk of PTS. So, the purpose of this systematic review is to assess the efficacy, safety and effectiveness of thrombolytic therapy in the treatment of acute DVT.

**Rationale**

Currently,the use of thrombolytic therapy as first-line therapy for acute DVT is not recommended in most treatment guidelines despite their use is appreciated through different studies. All studies included in this review are RCTs to maximize the quality of the results. 

## MATERIALS AND METHODS

 In this review, an attempt was made to include all published articles that were reported on the use of thrombolytic for acute deep venous thrombosis (DVT) by searching the PubMed and Google scholar electronic database. The following key words were used: thrombolytic, thrombolysis, fibrinolysis, fibrinolytics, therapy, tissue plasminogen activator and venous thrombosis.

**Eligibility criteria**

The following documents were not included: Unpublished documents, articles written in languages other than English, study design used other than RCT and articles published before 2000.

**Searching strategy**

Searching of articles from electronic database system of PubMed and Google Scholar was done from July 6 to July 13, 2018. A total of 760 articles were identified by systematic search strategy. After screening of the title and abstract using the predefined inclusion and exclusion criteria, 275 studies were retrieved for more detailed information. 518 articles were excluded for the following reasons: not written in English (n=44), not relevant to the topic (n=469), not consistent with study design (n=261, not RCT) and published before 2000 (n=5). Finally, 9 RCTs were included in this review.

**Key outcomes**

Efficacy, safety and effectiveness werethe key outcomes.

**Planned methods of analysis**

The validity of randomized trials with adequate reliability determined the adequacy of randomization and concealment of allocation, blinding of patients, health care providers, data collectors, and outcome assessors and extent of loss to follow-up (i.e. proportion of patients in whom the investigators were not able to ascertain outcomes.)

## Results

 The studies included in this systematic review were different types of interventions, ranging from non-pharmacologic management (compression stocking) to various pharmacotherapy managements (Urokinase, Alteplase, Heparinization, streptokinase, warfarin, enoxaparin, UFH and Actilyse). In studies which were tried to compare thrombolytic with standard management: almost all uses of heparin were followed by warfarin as standard therapy and most of the studies (five out of nine) use alteplase as thrombolytic agent during the study period. 

The data were analyzed from 7 countries, providing studylevel of 1309 participants from previously published studies. Surveys were broadly distributed across the three regions with more participants from Europe. Of 9 articles, 3 were conducted in Norway and the rest were carried out in China, Germany, Turkish, Egypt, the United States, and Brazil (Table 1). 

Regarding result presentation, three studies presented their data by comparing thrombolytic therapy with the standard anticoagulants treatment, two studies by dealing with post thrombotic complications after anticoagulants and thrombolytic therapy, and two other studies by concerning short- and long-term effectiveness of thrombolytic treatment, whereas the rest of the studies used catheter-directed thrombolysis for the treatment of DVT.

All publications were produced during the period 2000 and 2016. Most of the studies were conducted in a single study site (6 out of 9), and their results were presented by comparing standard anticoagulants with thrombolytic treatment. Five studies were done using catheter-directed thrombolytic therapy, while four of which employed systemic thrombolytic therapy. Three out of 9 studies compared standard treatment (anticoagulants) with thrombolytic therapy; two studies emphasized on the impacts of thrombolytic therapy in prevention of PTS, again 2 of which focused on short- and long-term results of thrombolytic treatment.

849 of 1309 patients were treated by thrombolytic therapy (urokinase, alteplase or streptokinase) and 460 of the patients were treated by standard anticoagulants (parenteral heparin followed by oral warfarin). 

**Figure:**
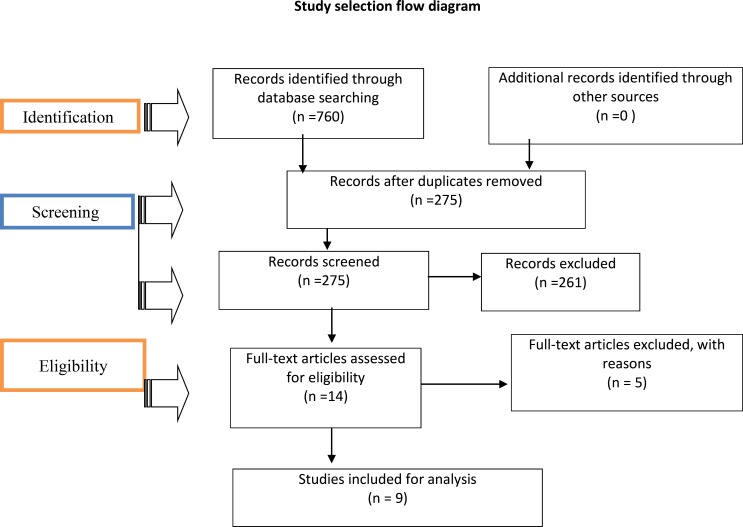


**Table 1:** Summary of studies included in the review

No	Year	Country	Site/ Sites	Subjects	Study purpose	Interventions/ medications	Outcome	citation
1	2016	China	1	106	Effect of CDT	Urokinase	Complication is high when giving in small saphenous vein.	12
2	2013	Turk	1	26	Efficacy of thrombolytic therapy	Alteplase	Thrombolytic therapy was successful for acute DVT	13
3	2000	Germany	1	250	short- and long-term efficacy of thrombolytictherapy	Heparinization, urokinase, streptokinase,	thrombolytic significantly reduced the number of closed veins	8
4	2009	Norway	19	118	Comparison of thrombolysis vs. anticoagulant	LMWH + warfarin Vs catheterized alteplase	Safety bleeding risk is higher with thrombolytic	14
5	2012	Norway	20	209	catheter-directed thrombolysis versus standard treatment	LMWH + warfarin Vs alteplase	PTS rate is lower in case of thrombolytic	15
6	2016	Norway	20	176	Thrombolytic for PTS	Alteplase	persistent and increased clinical benefit	16
7	2002	Egypt	1	35	Compare anti-coagulants and thrombolytic	LMWH + warfarin Vs streptokinase	thrombolysis obtained better patency and competencethan those treated with standard anticoagulation	17
8	2010	US	1	183	Compare the efficacy and safety of anti-coagulants plus thrombolytic with anti- coagulant alone	Enoxaparin/UFH + warfarin + tPA + compression stockings Vs Enoxaparin/UFH + warfarin + compression stockings	In patients with symptomatic proximal DVT, PEVI plusanticoagulation may be superior to anticoagulation— alone in the reduction of VTE andPTS	8
9	2007	Brazil	1	206	low-dose recombinant tissue-type plasminogen activator infusion in the treatment of iliofemoral DVT	Actilyse, UFH	They are effective in thrombolysis’ activity	18

CDT: Catheter-Directed Thrombolysis; DVT: Deep Venous Thrombosis;UFH: Unfractionated Heparin; LMWH: Low Molecular Weight Heparin; tPA: Tissue Plasmogen Activator; VTE: Venous Thrombo-Embolism ; PTS: Post Thrombotic Syndrome

## Discussion

 DVT treatment includes anticoagulant therapy, pharmacologic thrombolysis (systemic thrombolysis, flow-directed thrombolysis, and catheter-directed thrombolysis), percutaneous mechanical thrombectomy, surgical thrombectomy and physical therapy ^   3 ^. Current guideline of antithrombotic therapy for VTE disease suggests that acute lower extremity DVT patients are most likely to benefit from thrombolytic therapy due to its efficacy^13^^, ^^19^. 

Thrombolytic therapy has been showed very effective in reversing closed veins, improving patency rate and reducing reflux^8^^, ^^17^. Many studies agreed that lower dose of recombinant tissue plasminogen activators (tPA) was safe and effective in various forms of DVT^7^^, ^^18^^, ^^20^^, ^^21^ ^,^^   22 ^. Thrombolytics are less likely to cause complication in later stages of treatment compared with standard treatment which composed of heparin and warfarin therapy. One study observed that the most effective mechanism for thrombolysis was the penetration of the plasminogen activator into the thrombus, followed by activation of plasminogen that binds to fibrin during the clotting process^   2 ^.

The occurrence of PTS was lower [n=849 (8.3%)] in patients treated with thrombolytics ^   23 ^^,^^   15 ^. Similar study revealed that 20 % developed PTS after thrombolytic therapy, while 77 % developed PTS from anticoagulation therapy ^   19 ^. Rethrombosis was also lower among patients on thrombolytics (n=849, 2.4%) than standard management (n= 460, 39%)^15^^, ^^17^^, ^^19^^, ^^21^. A study on Short- and Long-Term Results After Thrombolytic Treatment of DVT, High-dose thrombolysis led to better rates of complete recanalization after seven days than loco-regional lysis^   19 ^. 

The addition of thrombolytics on DVT management was resulted in persistence and increased clinical benefits ^   24 ^. The incidence of VTE was also lower in patients treated with thrombolytic than anticoagulant alone ^18^^, ^^25^. However, considering the safety issue, thrombolytic therapy associated with major bleeding and PE in most patients compared with traditional treatment (10.4% and 4.1%), respectively, especially with higher doses the occurrences of such events are increased ^   16 ^. one study underlines that the use of thrombolytic needs further study and investigation to decide about their long-term effects^8^^, ^^15^. The utilization of these agents in the assessment of the quality of life in patients and their use specifically for endovascular thrombosis need further investigation (n=849 ,54%) compared to patients on anticoagulants (n=460 , 53%) ^   14 ^. 

One study reported increased rate of serious bleeding and PE after thrombolytic use ^   24 ^ and out of 12 patients receiving thrombolysis (9 systemic, 3 local); 9 patients on systemic treatment developed PE ^1^^, ^^2^. Furthermore, the study revealed that higher doses of thrombolytic were associated with serious adverse events (major bleeding and PE) and these agents can be resulted in better clinical outcome when given in catheter-directed route than systemic administration^21^^, ^^24^. Moreover, one study pointed out that these agents should only be considered in patients with high proximal DVT and lower risks of bleeding^   26 ^. 

## CONCLUSION

 The use of thrombolytic therapy offers potential advantages over the standard treatment of DVT by reducing the proportion of patients with chronic disabling leg symptoms (from PTS) by one-third in the longer term. However, the safety issues of these drugs in terms of risk of bleeding and PE require further investigation. 

## Abbreviations

CDT: Catheter-Directed Thrombolysis 

DVT: Deep Venous Thrombosis

LMWH: Low Molecular Weight Heparin 

PAI-1 Inhibitors: Inhibitors of Type-1 Plasminogen Activator Inhibitor 

PE: Pulmonary Embolism 

PEVI: Percutaneous Endo-Vascular Intervention

PTS: Post Thrombotic Syndrome

Rt-PA: Recombinant Tissue Plasminogen Activator

TAFIa: Thrombin Activatable Fibrinolysis Inhibitor

tPA: Tissue Plasminogen Activator

UFH: Unfractionated Heparin

VTE: Venous Thrombo-Embolism 

## Competing interests

 The authors declare that they have no competing interests.

## Funding

No funds have been received to conduct this study.
